# Pharmacogenomic Study Reveals New Variants of Drug Metabolizing Enzyme and Transporter Genes Associated with Steady-State Plasma Concentrations of Risperidone and 9-Hydroxyrisperidone in Thai Autism Spectrum Disorder Patients

**DOI:** 10.3389/fphar.2016.00475

**Published:** 2016-12-02

**Authors:** Sadeep Medhasi, Darawan Pinthong, Ekawat Pasomsub, Natchaya Vanwong, Nattawat Ngamsamut, Apichaya Puangpetch, Monpat Chamnanphon, Yaowaluck Hongkaew, Jirawat Pratoomwun, Penkhae Limsila, Chonlaphat Sukasem

**Affiliations:** ^1^Department of Pharmacology, Faculty of Science, Mahidol UniversityBangkok, Thailand; ^2^Division of Pharmacogenomics and Personalized Medicine, Department of Pathology, Faculty of Medicine Ramathibodi Hospital, Mahidol UniversityBangkok, Thailand; ^3^Laboratory for Pharmacogenomics, Somdech Phra Debaratana Medical Center, Ramathibodi HospitalBangkok, Thailand; ^4^Division of Virology, Department of Pathology, Faculty of Medicine Ramathibodi Hospital, Mahidol UniversityBangkok, Thailand; ^5^Yuwaprasart Waithayopathum Child and Adolescent Psychiatric HospitalSamut Prakarn, Thailand

**Keywords:** risperidone, autism spectrum disorders, pharmacogenomics, drug metabolizing enzymes, transporters

## Abstract

The present study sought to investigate the genetic variants in drug metabolizing enzyme and transporter (DMET) genes associated with steady-state plasma concentrations of risperidone among Thai autism spectrum disorder (ASD) patients. ASD patients taking risperidone for at least 1 month were enrolled for this pharmacogenomic study. Genotyping profile was obtained using Affymetrix DMET Plus array interrogating 1931 variants in 231 genes. Steady-state plasma risperidone and 9-hydroxyrisperidone were measured using liquid chromatography/tandem mass spectrometry assay. The final analysis included 483 markers for 167 genes. Six variants, *ABCB11* (c.3084A > G, c.^∗^420A > G, c.^∗^368G > A, and c.^∗^236G > A) and *ADH7* (c.690G > A and c.-5360G > A), were found to be associated with plasma concentrations of risperidone. 9-Hydroxyrisperidone and the total active-moiety levels were associated with six gene variants, *SCLO1B1* (c.-11187G > A and c.521T > C), *SLCO1B3* (c.334G > T, c.699A > G, and c.1557G > A), and *SLC7A5* c.^∗^438C > G. Polymorphisms in *UGT2B4* c.^∗^448A > G and *CYP2D6* (c.1661G > C, c.4180G > C, and c.-2178G > A) showed considerable but not significant associations with metabolic ratio. This pharmacogenomic study identifies new genetic variants of DMET genes in monitoring risperidone therapy.

## Introduction

Autism spectrum disorder (ASD) is a neurodevelopmental disorder typically diagnosed in early childhood, and is characterized by deficits in social and linguistic communication skills, along with repetitive and stereotyped behaviors. In addition, autism patients show symptoms like irritability, aggressive attitude toward other or property, anxiety, self-injurious behavior, hyperactivity, and severe temper tantrums (Ghosh et al., 2013; Bowers et al., 2015). Risperidone, an atypical antipsychotic, is approved by the Food and Drug Administration (FDA) to treat disruptive behaviors associated with autism in children and adolescents(Cohen et al., 2013) demonstrating antagonism at dopamine D_2_, serotonin 5HT_2A_, and adrenergic α_2_ receptors (Thyssen et al., 2010).

Plasma concentrations of risperidone and 9-hydroxyrisperidone vary among individuals, which in turn influences clinical responses and adverse-effects justifying the regular need to monitor plasma concentrations of risperidone, 9-hydroxyrisperidone, and the total active-moiety (Riedel et al., 2005; Locatelli et al., 2010). Genetic heterogeneity is commonly regarded as the main cause of this inter-individual variability. Pharmacogenomics explores the genetic causes of variability in drug responses, and aids in tailored therapy according to the individual’s genetic make-up (Collins et al., 2016).

Risperidone undergoes 9-hydroxylation producing 9-hydroxyrisperidone as its active metabolite. CYP2D6 is the key enzyme for the hydroxylation of risperidone, but CYP3A4 and CYP3A5 enzymes have also been reported to play minor roles in the metabolism of risperidone (Fang et al., 1999). 9-Hydroxyrisperidone and risperidone, which have similar pharmacological activities, are collectively termed as the total active-moiety. Nevertheless, studies have shown that risperidone and 9-hydroxyrisperidone differ from each other in terms of regulation of receptor-mediated signaling pathways (Clarke et al., 2013) and 5-HT_2A_/D_2_ affinity ratios with differences in their therapeutic efficacies or adverse effect profiles (Kozielska et al., 2012). Risperidone and 9-hydroxyrisperidone are substrates of *P*-glycoprotein, an adenosine triphosphate–binding cassette (ABC) superfamily transporter, and their dispositions and absorptions might be affected by the functional status of *P*-glycoprotein (Nakagami et al., 2005; Corena-McLeod, 2015).

Studies have reported the inter-individual variability in pharmacokinetic and pharmacodynamics of risperidone leading to unusual exposures, an occurrence of toxic effects, and therapeutic failure. Presence of single nucleotide polymorphisms (SNPs) in the genes encoding phase I and phase II drug-metabolizing enzymes (DME) alters the activity of enzymes resulting in four possible phenotypes: poor metabolizer (PM), intermediate metabolizer (IM), extensive metabolizer (EM), and ultrarapid metabolizer (UM; Zhou et al., 2009). Drug transporters are classified as influx solute carrier (SLC) family transporters and efflux ABC family transporters (Yiannakopoulou, 2013). A study analyzing the associations between drug metabolizing enzyme and transporter (DMET) polymorphisms and pharmacokinetic variability of risperidone found associations of polymorphisms in *CYP2D6*, *TPMT*, *ADRB1*, *VKORC1*, and *COMT* with pharmacokinetic parameters (Cabaleiro et al., 2014). *ABCB1* 3435C>T genotypes had significant effect on maximum concentration observed (*C*_max_) of risperidone and the total active-moiety, but when *ABCB1* 3435C>T genotypes were combined with *CYP2D6^∗^10/^∗^10* genotype carrying subjects, significant differences were observed in *C*_max_, the area under the curve (AUC) of risperidone, and the total active-moiety as well (Yoo et al., 2011). Several polymorphisms have been studied emphasizing the influence of allelic variations among metabolic enzymes and transporters in the disposition of risperidone and its phenotypes. An association study of risperidone and 9-hydroxyrisperidone plasma levels with *CYP2D6* polymorphisms in ASD patients found a significant influence of *CYP2D6* variants on plasma risperidone concentrations which provides evidence of genetic variations of drug exposure and safety (Vanwong et al., 2016a). Previous studies also reported the influence of polymorphisms in DMET genes, including *CYP2D6*, *CYP3A4*, *CYP3A5*, *ABCB1*, *COMT*, and *VKORC1* on the plasma concentrations of risperidone, 9-hydroxyrisperidone, and the total active-moiety (Jovanovic et al., 2010; Xiang et al., 2010; Suzuki et al., 2012; Cabaleiro et al., 2014). The present authors previously reported the prevalence of clinically important DMET SNPs in Thai ASD patients (Medhasi et al., 2016).

The influence of *CYP2D6*, *CYP3A5*, and *ABCB1* gene variants on plasma levels of risperidone, 9-hydroxyrisperidone, and the total active-moiety clearly argues for the need of pharmacogenetic testing and therapeutic drug monitoring (TDM) during risperidone therapy (Jovanovic et al., 2010). The association between genetic variants of DMET genes and steady-state plasma concentrations of risperidone, 9-hydroxyrisperidone, total active-moiety, and metabolic ratio remains unclear. The objective of the present study was to present the results of the association between genetic variants of the DMET genes and steady-state plasma concentrations of risperidone and its metabolite using the Affymetrix DMET Plus genotyping microarrays.

## Materials and Methods

### Patients

Patients eligible for this study included male and female adolescents with ASD diagnosed according to the *Diagnostic and Statistical Manual of Mental Disorders, Fourth Edition* (DSM-IV) criteria and being treated with risperidone for at least 4 weeks prior to blood sample collection. Subjects enrolled were outpatients from the Yuwaprasart Waithayopathum Child and Adolescent Psychiatric Hospital, Samut Prakarn, Thailand for this retrospective pharmacogenomic study. Only patients having complete data on doses and duration of risperidone were recruited for this study. Dosing of the risperidone was flexible depending on response or adverse events. Risperidone dosing followed the guidelines where the starting dose of risperidone for the treatment of irritability associated with autistic disorder is 0.25 mg/day for patients weighing <20 kg and 0.5 mg/day for patients weighing ≥20 kg, with the target dose of 0.5 mg/day (<20 kg) and 1 mg/day (≥20 kg); effective dose range, 0.5–3 mg/day (Risperdal prescribing information, revised version 2014). Patient compliance was assessed and confirmed by the nursing staff.

Medications co-administered along with risperidone were recorded. In the case of the co-occurring attention deficit/hyperactivity disorder, stimulants were allowed to continue, provided no changes were made during the study. The exclusion criteria included patients with severe physical disabilities and currently being treated with other antipsychotics that could interfere with the pharmacokinetics of risperidone. This study was carried out in accordance with the principles of the Declaration of Helsinki, and was approved by the Ramathibodi Ethics Committee (Bangkok, Thailand). The parents of all patients included in the study received an explanation of the purpose and experimental procedures of the study. A written informed consent was obtained from the parents or guardians of the patients before the study.

### Blood Sampling and Measurements of Risperidone and 9-Hydroxyrisperidone

Blood samples were drawn from the patients during 08:00–09:00 AM prior to the next dose of risperidone intake to determine the steady-state plasma trough concentrations (Css) of risperidone and 9-hydroxyrisperidone. Plasma samples were collected into EDTA tubes and stored at -80°C until analyzed at the laboratory for TDM in the Faculty of Medicine, Ramathibodi Hospital (Bangkok, Thailand).

The quantitative determinations of Css of risperidone and 9-hydroxyrisperidone were performed using a modified validated liquid chromatography coupled with the tandem mass spectrometry (LC-MS/MS) method (Vanwong et al., 2016b). The instrument consisted of an Agilent 1260 HPLC system (Agilent Technologies, Santa Clara, CA, USA) coupled to an API 3200^TM^ (SCIEX) mass spectrometer. The mass-to-charge ratio (m/z) applied for ion monitoring was 411→191 for risperidone, 428→207 for 9-hydroxyrisperidone, and 328→270 for the internal standard clozapine.

The values of intra-assay and inter-assay coefficients of variation were less than 9% for risperidone and less than 6% for 9-hydroxyrisperidone. The average accuracy was 98–110% for risperidone and 95–105% for 9-hydroxyrisperidone. The lower limit of quantifications (LLOQ) was 0.2 and 0.5 ng/ml for risperidone and 9-hydroxyrisperidone.

### DMET Plus Array and SNP Genotyping and Quality Control

Genomic DNA was extracted from EDTA-whole blood using the MagNA Pure Compact System (Hoffman-La Roche Ltd., Basel, Switzerland). Genotyping of SNPs was performed using molecular inversion probes (MIP) technology on the Affymetrix DMET^TM^ Plus GeneChip microarray platforms (Affymetrix Inc., Santa Clara, CA, USA). The Affymetrix DMET^TM^ Plus microarray enables the genotyping of 1,936 genetic markers (1,931 SNPs and 5 CNVs) across 231 genes having functional significance in drug pharmacokinetics (Bonifaz-Pena et al., 2014). The PCR product from MIP technology undergoes enzymatic fragmentation and then hybridization to the DMET microarray for SNP discrimination and genotyping (Hardenbol et al., 2003; Burmester et al., 2010). Washing and staining of DMET arrays were performed using Affymetrix fluidic stations and scanned by the Affymetrix GeneChip^®^ Scanner 3000 (Affymetrix Inc, Santa Clara, CA, USA). The genotyping profiles of DMET SNPs were generated with Affymetrix DMET Console software^®^ (version 1.3).

Genotyping of the previously associated *CYP2D6* variants, 1846G > A, rs3892097; 100C > T, rs1065852; and 2988G > A, rs28371725, with risperidone pharmacokinetics (Scordo et al., 1999; Yasui-Furukori et al., 2003; Riedel et al., 2005; Vanwong et al., 2016a) which failed the quality control (QC) in microarray analyses were identified using the PCR-based TaqMan allele discrimination method (Applied Biosystems, Carlsbad, CA, USA) for further analysis.

The QC check on the genotyping data was performed using the R package “GenABEL” and Haploview software (Barrett et al., 2005). Individuals with a <90% genotyping call rate were excluded and SNPs with more than 5% missing genotypes were excluded. Also, SNPs with minor allele frequency (MAF) < 0.05, a deviation from Hardy–Weinberg equilibrium (HWE) at *P* > 0.001, and variants of chromosome X were discarded from further analysis.

### Data Analyses and Statistics

The null hypothesis for this genetic association study was that genetic variants of DMET genes are not associated with Css of risperidone and 9-hydroxyrisperidone. Results are expressed as median and the range, unless specified otherwise. The Shapiro–Wilk test was used to assess the normality of the plasma concentrations of risperidone and 9-hydroxyrisperidone. Q-Q plots, multidimensional scaling (MDS) plot, and association analysis of DMET markers with plasma concentrations of risperidone, 9-hydroxyrisperidone, the total active-moiety, and metabolic ratio were estimated using Cochran–Armitage test for trends in the GenABEL package of R version 3.1.2 (Aulchenko et al., 2007). The *P*-value adjusted after Bonferroni correction was set at 2.5 × 10^-5^. Being an exploratory study, however, genetic markers were selected as notable ones in the association analysis with a *P*-value less than 0.01, corrected for possible inflation due to some degree of stratification. The demographic, phenotypic measurements and linear regression analyses were calculated using the SPSS (version 17.0). *P <* 0.05 was considered significant in the linear regression analysis. Pairwise linkage disequilibrium analysis was performed using Haploview 4.2.

## Results

### Characteristics of the Study Cohort and Selection of DMET Markers

After screening the 104 individuals for missing data, 103 patients passed the genotyping call rate threshold set at 90%. In addition, one patient was excluded because of being too highly identical by descent (IBS) (≥0.95). **Table 1** shows the demographic and clinical characteristics of the 102 autistic patients treated with risperidone. The study population had a median (range) age of 8.8 (3.4–18.6) years which was predominantly boys (85.3%). The median risperidone dose was 0.5 (range: 0.2–4) mg/day. Patients had a median range of risperidone duration of treatment of 41.62 (1.03–152.97) months. The percentage of the patients treated with only risperidone was 61.76%. Concomitant medication use among the patients was 16.67% prevalently using methylphenidate followed by 15.69% patients who were using valproic acid. Other concomitant medicines included fluoxetine, pyrithioxine, and sertraline.

**Table 1:** Demographic and clinical characteristics of the ASD patients.

Characteristic	*n* = 102
Age, years (range)	8.8 (3.4–18.6)
Sex:	
Male	87 (85.3%)
Female	15 (14.7%)
Risperidone treatment	
Risperidone dose, mg/day (range)	0.5 (0.2–4)
Risperidone duration, months (range)	41.62 (1.03–152.97)
Risperidone single medication, *n* (%)	63 (61.76)
Concomitant treatments, n (%)	
Methylphenidate	17 (16.67)
Valproic acid	16 (15.69)
Methylphenidate + valproic acid	3 (2.94)
Methylphenidate + fluoxetine	1 (0.98)
Pyrithioxine	1 (0.98)
Sertraline	1 (0.98)
Median of plasma drug concentrations, ng/ml (IQR)	
Risperidone	0.83 (2.22)
9-hydroxyrisperidone	6.75 (7.42)
Total active-moiety	8.48 (8.34)
Median metabolic ratio (IQR)	0.08 (0.31)


*ASD, autism spectrum disorder; IQR, interquartile range.*

After filtering 1931 SNP markers with the QC parameters, 171 (8.9%) markers were removed owing to an overall genotyping call rate of less than 95%. From the remaining 1760 markers, 1229 markers were removed from further analysis because their MAF was less than 5%. Furthermore, 46 markers on the X chromosome and two markers with HWE *P*-value < 0.001 were excluded as well. As a result, 483 (25%) markers of 1931 markers were included for final analysis. A MDS plot did not show any clear clusters which means that no population stratification exists and also there were no differences in allele frequencies between populations (**Supplementary Figure S1**).

### Plasma Concentrations of Risperidone and Its Metabolite

The median (IQR) trough levels of risperidone and 9-hydroxyrisperidone measured 12 h after the last risperidone administration were 0.83 (2.22) ng/ml and 6.75 (7.42) ng/ml. The median (IQR) plasma concentration of the total active moiety was 8.48 (8.34) ng/ml as shown in **Table 1**. The median (IQR) metabolic ratio was 0.08 (0.31).

### Association of DMET SNPs in Steady-State Plasma Concentrations of Risperidone and 9-Hydroxyrisperidone

The DMET SNPs showing significant associations with plasma concentrations of risperidone and its metabolite with a minimum *P*-value less than 0.01 included 22 SNPs of seven genes from the GeneChip microarray analyses; **Tables 2**–**5**. The SNPs showing a suggestive evidence of association, at *P* < 0.05, are shown in Supplementary Tables S1–S4.

**Table 2:** Top SNPs associated with steady-state plasma risperidone concentrations (Sample size = 102).

SNP rsID	Marker name	Amino acid change	Chromosome	Cytoband	*P*-values
rs497692	ABCB11 c.3084A > G	A1028A	2	q24.3	0.0062
rs496550	ABCB11 c.^∗^420A > G	3′UTR	2	q24.3	0.0063
rs495714	ABCB11 c.^∗^368G > A	3′UTR	2	q24.3	0.0068
rs971074	ADH7 c.690G > A	R230R	4	q23	0.0092
rs1442477	ADH7 c.-5360G > A	Promoter	4	q23	0.0092
rs473351	ABCB11 c.^∗^236G > A	3′UTR	2	q24.3	0.0094


*SNPs, single-nucleotide polymorphisms; UTR, untranslated region.*

**Table 3:** Top SNPs associated with steady-state plasma 9-hydroxyrisperidone concentrations (Sample size = 102).

SNP rsID	Marker name	Amino acid change	Chromosome	Cytoband	*P*-values
rs4149015	SLCO1B1 c.-11187G > A	Promoter	12	p12.2	0.0001
rs1060253	SLC7A5 c.^∗^438C > G	3′UTR	16	q24.2	0.0005
rs4149117	SLCO1B3 c.334G > T	A112S	12	p12.2	0.0021
rs7311358	SLCO1B3 c.699A > G	I233M	12	p12.2	0.0021
rs2053098	SLCO1B3 c.1557G > A	A519A	12	p12.2	0.0021
rs4149056	SLCO1B1 c.521T > C	V174A	12	p12.1	0.0090


**Table 4:** Top SNPs associated with steady-state plasma total active-moiety concentrations (Sample size = 102).

SNP rsID	Marker name	Amino acid change	Chromosome	Cytoband	*P*-values
rs4149015	SLCO1B1 c.-11187G > A	Promoter	12	p12.2	0.0001
rs1060253	SLC7A5 c.^∗^438C > G	3′UTR	16	q24.2	0.0007
rs4149117	SLCO1B3 c.334G > T	A112S	12	p12.2	0.0015
rs7311358	SLCO1B3 c.699A > G	I233M	12	p12.2	0.0015
rs2053098	SLCO1B3 c.1557G > A	A519A	12	p12.2	0.0015
rs4149056	SLCO1B1 c.521T > C	V174A	12	p12.1	0.0081


**Table 5:** Top SNPs associated with risperidone/9-hydroxyrisperidone metabolic ratio (Sample size = 102).

SNP rsID	Marker name	Amino acid change	Chromosome	Cytoband	*P*-values
rs1131878	UGT2B4 c.^∗^448A > G	3′UTR	4	q13.2	0.0022
rs1058164	CYP2D6 c.1661G > C	V136V	22	q13.2	0.0030
rs1135840	CYP2D6 c.4180G > C	S486T	22	q13.2	0.0046
rs28360521	CYP2D6 c.-2178G > A	5′UTR	22	q13.2	0.0050


**FIGURE 1:**
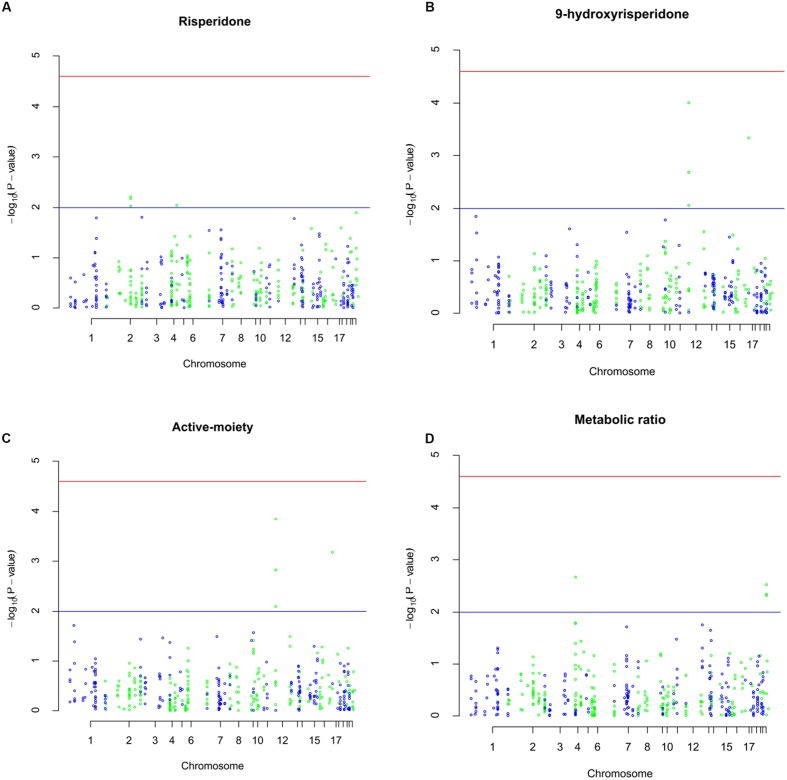
**Manhattan plots of associations of plasma concentrations of risperidone, 9-hydroxyrisperidone, total active-moiety, and metabolic ratio with 483 drug metabolizing enzyme and transporter (DMET) single nucleotide polymorphisms (SNPs).** The horizontal *x*-axis represents the chromosomal positions; the vertical *y*-axis represents –log_10_*P*-values from the linear regression. The red horizontal line represents the significance level of *P* = 2.5 × 10^-5^ after Bonferroni correction. The horizontal blue line represents the *P*-value of 0.01. **(A)** Manhattan plot showing the significance of DMET SNPs with plasma concentrations of risperidone. **(B)** Manhattan plot showing the significance of DMET SNPs with plasma concentrations of 9-hydroxyrisperidone. **(C)** Manhattan plot showing the significance of DMET SNPs with plasma concentrations of total-active moiety. **(D)** Manhattan plot showing the significance of DMET SNPs with metabolic ratio.

The top significant findings of DMET SNPs associated with steady-state plasma concentrations of risperidone are shown in **Table 2** and **Figure 1A**. Of all the DMET SNPs, six SNPs were found to be associated with plasma concentrations of risperidone (*P* < 0.01). Analysis showed 4 *ABCB11* SNPs, including c.3084A > G (rs497692), c.^∗^420A > G (rs496550), c.^∗^368G > A (rs495714), and c.^∗^236G > A (rs473351), and 2 *ADH7* SNPs which included c.690G > A (rs971074) and c.-5360G > A (rs1442477) that had significant associations.

The 9-hydroxyrisperidone plasma concentrations correlated significantly with the DMET genetic markers (*P* < 0.01) and are illustrated in **Table 3** and **Figure 1B**. A total of six genetic variations among the SLC transporter family were associated with the plasma concentrations of 9-hydroxyrisperidone. Of the *SLCO1B1* polymorphisms, c.-11187G > A (rs4149015) and c.521T > C (rs4149016) had significant associations (*P* < 0.01). Three variants of *SLCO1B3*, including c.334G > T (rs4149117), c.699A > G (rs7311358), and c.1557G > A (rs2053098) that influenced the plasma concentrations of 9-hydroxyrisperidone. *SLC7A5* c.^∗^438C > G (rs1060253) also showed considerable influence in the plasma concentrations of the metabolite.

As **Table 4** and **Figure 1C** shows, identical DMET SNPs were associated with plasma concentrations of the total active-moiety as were identified for the associations with 9-hydroxyrisperidone. The DMET SNPs significantly influenced the metabolic ratio (*P* < 0.01, **Table 5**; **Figure 1D**) that involved four metabolic enzyme variants. *UGT2B4* c.^∗^448A > G (rs1131878), *CYP2D6* c.1661G > C (rs1058164), *CYP2D6* c.4180G > C (rs1135840), and *CYP2D6* c.-2178G > A (rs28360521) were the four metabolic enzyme polymorphisms highly associated with metabolic ratio.

### Association of CYP2D6 SNPs in Steady-State Plasma Concentrations of Risperidone and 9-Hydroxyrisperidone Using TaqMan Assay

The relationships between the *CYP2D6* variants (rs3892097, rs1065852, and rs28371725) and the plasma concentrations of risperidone, 9-hydroxyrisperidone, the total active-moiety, and the metabolic ratio are shown in **Table 6**. A significant influence in the plasma concentration of risperidone (*P* = 0.009) was observed with *CYP2D6* c.2988G > A (rs28371725), but no significance was found in the plasma concentration of 9-hydroxyrisperidone, the total active-moiety, and metabolic ratio. There was significant influence of *CYP2D6* c.100C > T (rs1065852) in the metabolic ratio (*P* = 0.004), whereas no influence was found for plasma concentrations of risperidone, 9-hydroxyrisperidone, and the total active-moiety. *CYP2D6* c.1846G > A (rs3892097) did not show any associations with the plasma concentrations of risperidone, 9-hydroxyrisperidone, the total-active-moiety, and the metabolic ratio.

**Table 6:** Relationship between *CYP2D6* variant alleles and the plasma concentrations of risperidone, 9-hydroxyrisperidone, total active-moiety, and metabolic ratio (Sample size = 102).

*CYP2D6* marker	*P*-values			
	
	Risperidone	9-hydroxyrisperidone	Total active-moieties	Risperidone/9-hydroxyrisperidone
c.1846G > A, rs3892097	0.39	0.9	0.22	0.37
c.100C > T, rs1065852	0.12	0.14	0.25	0.004^∗^
c.2988G > A, rs28371725	0.009^∗^	0.9	0.74	0.26


*^∗^*P*-value was considered significant.*

### Linkage Disequilibrium Analysis

**FIGURE 2:**
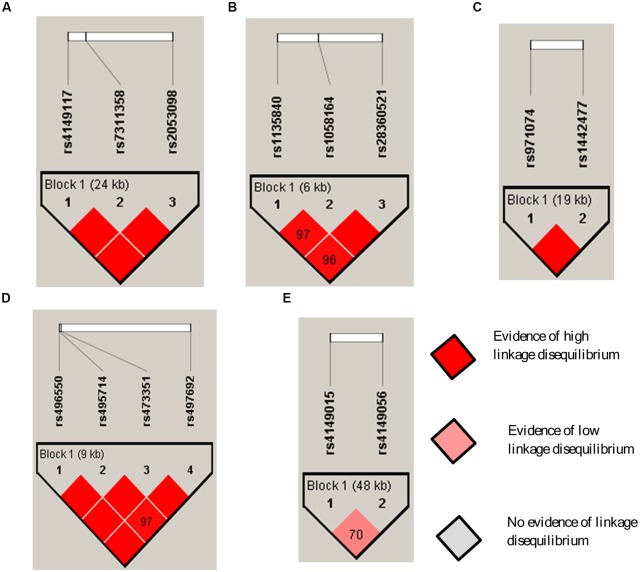
**Haploview linkage disequilibrium map of SNPs across DMET genes:** **(A)** *SLCO1B3*; **(B)** *CYP2D6*; **(C)** *ADH7*; **(D)** *ABCB11*; and **(E)** *SLCO1B1*. Pairwise linkage disequilibrium (D′) values are given in blocks for each SNP combination. Dark red shading denotes D′-values > 0.9, and empty dark red blocks indicate D′values of 1.0.

Linkage disequilibrium analysis of 14 SNPs across five genes is shown in **Figure 2**. Strong linkage disequilibria (D′-values > 0.9) were observed between *SLCO1B3* rs4149117, rs7311358, and rs2053098; *CYP2D6* rs1058164, rs1135840, and rs28360521; *ADH7* rs971074 and rs1442477; and *ABCB11* rs496550, rs497692, rs495714, and rs473351. Linkage disequilibrium was not strong across the *SLCO1B1* rs4149015 and rs4149056.

## Discussion

In this study, the associations of DMET genetic markers with steady-state plasma concentrations of risperidone and 9-hydroxyrisperidone among Thai ASD risperidone-treated patients using Affymetrix DMET Plus microarray platform were evaluated. The major findings from this exploratory pharmacogenomic investigation were the abilities of the genetic markers involved in drug metabolism and transport to predict inter-individual variability in steady-state plasma concentrations of risperidone and its active metabolite, 9-hydroxyrisperidone. It showed that six polymorphisms (4 *ABCB11* variants and 2 *ADH7* variants) were strongly associated with plasma concentrations of risperidone; six *SLC* polymorphisms (3 *SLCO1B3* variants, 2 *SLCO1B1* variants, and 1 *SLC7A5* variant) were strongly associated with plasma concentrations of 9-hydroxyrisperidone and the total active-moiety; and four polymorphisms (3 *CYP2D6* variants and 1 *UGT2B4* variant) were strongly associated with metabolic ratio.

With the increased recognition of the role of transporters in drug disposition with pharmacologically important allelic variability, both ABC and SLC transporters have been the scope of analysis in the present study. These results showed that rs497692, rs496550, rs495714, and rs473351 in *ABCB11* were associated with plasma concentrations of risperidone. *ABCB11* is a member of the ABC transporter group, subfamily B, member 11, and is also known as sPgp (sister of P-glycoprotein)^1^. Although *ABCB11* has been studied extensively in other conditions, the report herein found new SNPs of *ABCB11* that are associated with plasma concentrations of risperidone. In the study, genes and SNPs that do not have direct interactions with risperidone and its metabolite have been discovered and showed associations with plasma concentrations of risperidone and its metabolite. SNPs related to *ADH7* (rs971074 and rs1442477) showed an association with plasma concentrations of risperidone. ADH7 is a class IV alcohol dehydrogenase which metabolizes ethanol to acetaldehyde (Jairam and Edenberg, 2014). It can then be concluded that there is a possible role for multiple genetic variants which previously lacked evidence of pharmacogenomic associations and known biological pathways and that now lead to phenotypic outcomes similar to the established ones. This explains the advantage of microarray technology where an *a priori* assumption is avoided and an established physiological network among several genes in the body is recognized (Nigam, 2015; Arbitrio et al., 2016). The mechanistic functional effects of previously unrelated genes, however, should be further investigated in an independent sample set to reveal the linking molecular pathways.

Other top SNPs associated with plasma concentrations of 9-hydroxyrisperidone and the total active-moiety were SLC transporter variants. A previous study reported the influence of *SLC6A4* in sexual dysfunction and weight gain among risperidone treated patients (Almoguera et al., 2013). The present study, however, found SNPs among *SLCO1B1*, *SLCO1B3*, and *SLC7A5* were associated with plasma concentrations of 9-hydroxyrisperidone and the total active-moiety. *CYP2D6* polymorphisms influenced the plasma risperidone to 9-hydroxyrisperidone ratio in this study which suggests the functional role of CYP2D6 on a genetic basis. Among the Phase II enzymes, SNP rs1131878 in *UGT2B4* was shown to affect the risperidone metabolic ratio. UDP-glucuronyltransferase (UGT) enzymes catalyze the glucuronidation reactions and are an important aspect for variability in pharmacokinetics and clinical responses of antipsychotic agents (Kiang et al., 2005).

This pharmacogenomic study identifies newer variants of DMET gene polymorphisms with greater susceptibility to alter plasma concentrations of risperidone, 9-hydroxyrisperidone, total active-moiety, and metabolic ratio. Notably, this study did not find any significant associations among the variants previously reported to influence risperidone and 9-hydroxyrisperidone plasma concentrations. Polymorphisms among the main metabolic polymorphic enzyme *CYP2D6* and the major transporter protein *ABCB1* did not correlate with the plasma concentrations of risperidone or 9-hydroxyrisperidone as has been reported in earlier studies among the schizophrenic patients (Roh et al., 2001; Jovanovic et al., 2010). This finding implicates the limited importance of *CYP2D6* and *ABCB1* polymorphisms for the plasma concentrations of risperidone and 9-hydroxyrisperidone. *ABCB1* 3435C > T polymorphisms, however, were significantly associated with pharmacokinetic parameters (*C*_max_, AUC, and MR) of risperidone among individuals who were carriers of *CYP2D6^∗^10/^∗^10* (Yoo et al., 2011). The reduced function variant *CYP2D6^∗^10* which is highly prevalent among Asian populations and associated with plasma concentrations of risperidone (Roh et al., 2001; Vanwong et al., 2016a) failed to pass the QC in the microarray assay. To make these results more conclusive, *CYP2D6* variants (rs3892097, rs1065852, and rs28371725) that showed significant influence in pharmacokinetics of risperidone in previous studies were genotyped using the TaqMan assay. The assay found the involvement of *CYP2D6* variant alleles in the metabolism of risperidone. The presence of a strong relationship between *CYP2D6* variants and metabolism of risperidone in both microarray and TaqMan assays suggests the important role of *CYP2D6* genotyping during risperidone treatment.

A cautious approach for the implementation of the information from this study is recommended as many SNPs did not pass the QC step and these SNPs could not be analyzed for associations with plasma concentrations of risperidone and 9-hydroxyrisperidone. A majority of the SNPs were discarded due to the QC filtering criteria of MAF < 0.05. It is likely that rare variants affecting the plasma concentration of risperidone and 9-hydroxyrisperidone might have been missed. Due to the lack of sample size, only the SNPs with a MAF > 0.05 were included and the possibility of false-positive findings was avoided.

The median risperidone dose was relatively lower (0.5 mg/day) along with plasma levels of the total active-moiety (8.48 ng/ml) which are lower than the values reported earlier causing extrapyramidal side effects (EPS; Spina et al., 2001). Linear regression analysis showed a significant influence of the risperidone dose in the plasma concentration of risperidone, 9-hydroxyrisperidone, and the total active-moiety but no effect on the metabolic ratio. The duration of risperidone therapy did not affect the plasma levels of risperidone, 9-hydroxyrisperidone, active-moiety, and metabolic ratio. Age affected the plasma level of 9-hydroxyrisperidone, the total active-moiety, and metabolic ratio. These findings suggest the possible variables responsible for interindividual variations in plasma concentrations of risperidone and its metabolite. Similar findings were reported in an earlier study with a similar effect of age on the plasma concentrations of the total active-moiety (Aichhorn et al., 2005).

This study has limitations. The lack of validation of the most significant (*P* < 0.01) DMET variants and association of steady-state plasma concentrations with drug response limits the significance of the study. These results should be verified and clinically interpreted in an independent cohort to avoid the risk of false-positive findings. Also, none of the variants met Bonferroni cutoff value. The exploratory nature of this study, however, suggests the preliminary results to be taken into consideration for further correlation studies. Another limitation is the absence of genomic control which could have false-negative conclusions. The lack of independent validation creates a space for other causative factors, such as age, dosage, and environmental interactions to be implicated for the influence on plasma concentrations of risperidone and its metabolite.

Despite limitations, therapeutic implications from these findings may aid in avoiding toxic plasma levels of risperidone and its metabolite in long term treatment. This pharmacogenomic study provides a mechanistic understanding for individual differences in pharmacokinetics of drugs and may have an outstanding application in the improved personalized treatment of autistic patients in risperidone dosing, efficacy, and side effects. This study opens up the prospect for association of DMET genetic variants with treatment response and adverse effects in future studies. All the patients involved in this study were unrelated which potentially avoided the bias of genotype within families being over-represented.

In summary, this study provides a pharmacogenomic approach to further investigate the DMET genetic variants which influence plasma concentrations of risperidone and its metabolite. The treatment of ASD should be based on genetic factors making the challenge of psychopharmacological treatment more efficacious with fewer adverse events. A caution must be exercised, however, in transferring the pharmacogenomic data into clinical settings unless the genotype-phenotype link is clearly validated.

## Author Contributions

SM, NV, and CS wrote the manuscript; CS, DP, PL, SM, and AP designed the research study; SM, NV, MC, YH, and JP performed the microarray, genotyping and laboratory part; NN and PL diagnosed and recruited the subjects; SM, CS, and EP analyzed the data.

## Conflict of Interest Statement

The authors declare that the research was conducted in the absence of any commercial or financial relationships that could be construed as a potential conflict of interest.

## Footnotes

1 http://www.ncbi.nlm.nih.gov/gene/8647

## Supplementary Material

**FIGURE S1 | Multidimensional scaling (MDS) plot of adjacent MDS components.** The plot shows absence of clear population stratification.
