# Supplementary figures and images for: Pharmacogenomic Study Reveals New Variants of Drug Metabolizing Enzyme and Transporter Genes Associated with Steady-State Plasma Concentrations of Risperidone and 9-Hydroxyrisperidone in Thai Autism Spectrum Disorder Patients
Source: Front Pharmacol. 2016 Dec 2;7:475. doi: 10.3389/fphar.2016.00475 (PMC5147413; doi:10.3389/fphar.2016.00475)

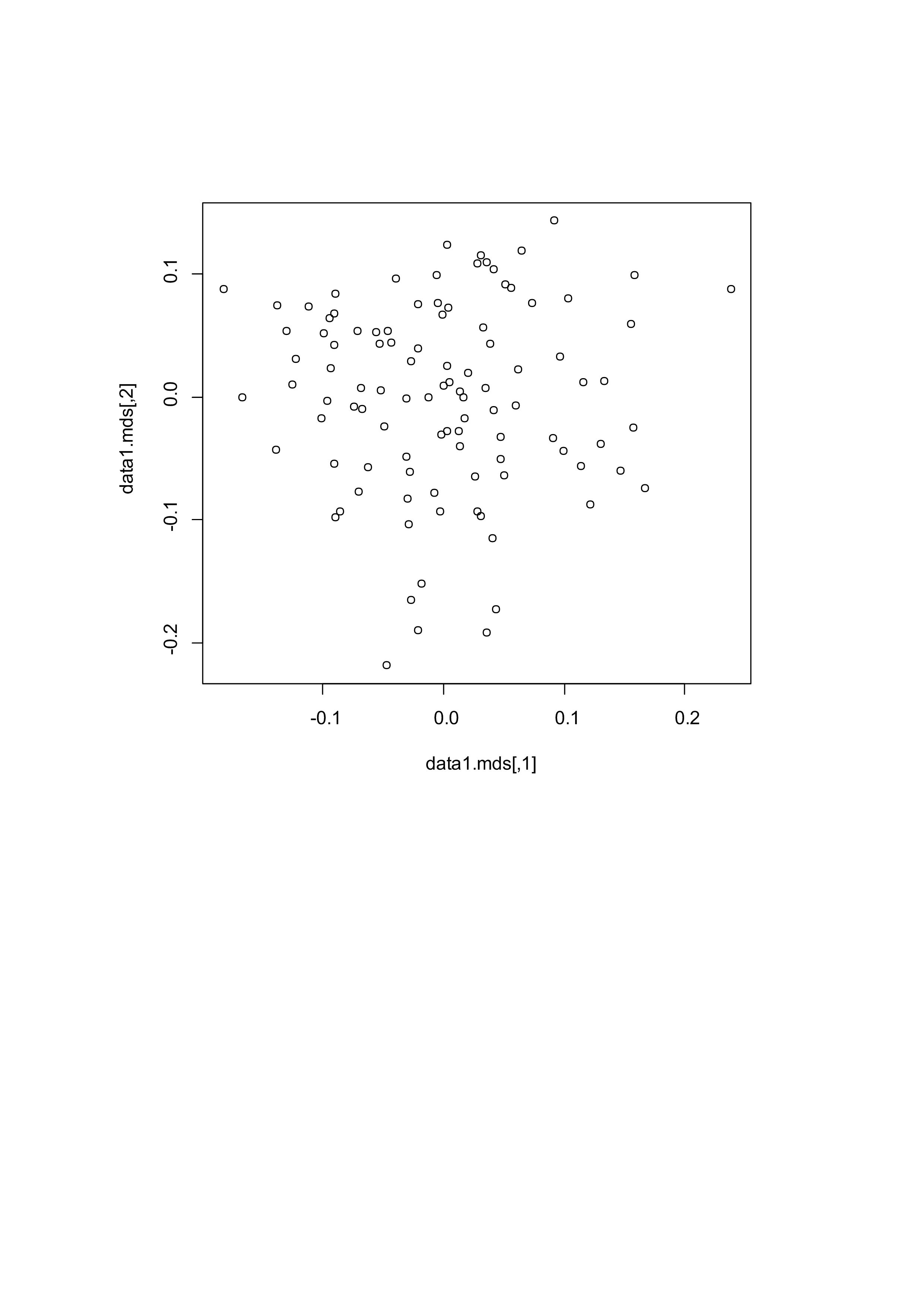

Supplement: Supplementary file 1 [file Image_1.TIF]
