# Supplementary material for: Pharmacogenomic Study Reveals New Variants of Drug Metabolizing Enzyme and Transporter Genes Associated with Steady-State Plasma Concentrations of Risperidone and 9-Hydroxyrisperidone in Thai Autism Spectrum Disorder Patients
Source: Front Pharmacol. 2016 Dec 2;7:475. doi: 10.3389/fphar.2016.00475 (PMC5147413; doi:10.3389/fphar.2016.00475)
Supplement: Supplementary file 2 [file Table_1.DOCX]

**Supplementary Table S1.** Top SNPs associated with steady-state plasma risperidone concentrations (Sample size = 102)**;** *P*<0.05

| SNP rsID | Marker name | Chromosome | Marker position | *P* values |  |
| --- | --- | --- | --- | --- | --- |
| rs497692 | ABCB11 c.3084A>G(A1028A) | 2 | 169789016 | 0.0062 | |
| rs496550 | ABCB11 c.*420A>G | 2 | 169779712 | 0.0063 | |
| rs495714 | ABCB11 c.*368G>A | 2 | 169779764 | 0.0068 | |
| rs971074 | ADH7 c.690G>A(R230R) | 4 | 100341861 | 0.0092 | |
| rs1442477 | ADH7 c.-5360G>A(rs1442477) | 4 | 100361787 | 0.0092 | |
| rs473351 | ABCB11 c.*236G>A | 2 | 169779896 | 0.0094 | |
| rs1058164 | CYP2D6 c.1661G>C(V136V) | 22 | 42525132 | 0.0127 | |
| rs3856806 | PPARG c.1431C>T(H477H) | 3 | 12475557 | 0.0157 | |
| rs7877 | FMO1 c.*207C>T | 1 | 171254890 | 0.0165 | |
| rs1801243 | ATP7B c.1216T>G(S406A) | 13 | 52548140 | 0.0171 | |
| rs3786362 | TYMS c.381A>G(E127E) | 18 | 662247 | 0.0261 | |
| rs4646285 | SLC10A1 c.225G>A(T75T) | 14 | 70263648 | 0.0264 | |
| rs2230028 | ABCB4 c.1954A>G(R652G) | 7 | 87056176 | 0.0282 | |
| rs2066853 | AHR c.1661G>A(R554K) | 7 | 17379110 | 0.0295 | |
| rs2242048 | SLC28A1 c.1368G>A(Q456Q) | 15 | 85478410 | 0.0341 | |
| rs2281593 | GSTA3 c.272+116C>T | 6 | 52767028 | 0.0379 | |
| rs2231142 | ABCG2 c.421C>A(Q141K) | 4 | 89052323 | 0.0381 | |
| rs8025045 | SLC28A1 c.*141G>T | 15 | 85488572 | 0.0383 | |
| rs6068816 | CYP24A1 c.9028G>A(T248T) | 20 | 52781091 | 0.0412 | |
| rs1126692 | FMO1 c.1188A>G(V396V) | 1 | 171252287 | 0.0416 | |
| rs4148805 | ABCB4 c.-1584C>T | 7 | 87106365 | 0.0419 | |
| rs742350 | FMO1 c.747C>T(T249T) | 1 | 171250044 | 0.0421 | |
| rs4148808 | ABCB4 c.-1014A>G | 7 | 87105795 | 0.0447 | |
