# Supplementary material for: Pharmacogenomic Study Reveals New Variants of Drug Metabolizing Enzyme and Transporter Genes Associated with Steady-State Plasma Concentrations of Risperidone and 9-Hydroxyrisperidone in Thai Autism Spectrum Disorder Patients
Source: Front Pharmacol. 2016 Dec 2;7:475. doi: 10.3389/fphar.2016.00475 (PMC5147413; doi:10.3389/fphar.2016.00475)
Supplement: Supplementary file 4 [file Table_3.DOCX]

**Supplementary Table S3.** Top SNPs associated with steady-state plasma active-moiety concentrations (Sample size = 102); *P*<0.05

| SNP rsID | Marker name | Chromosome | Marker position | *P* values |
| --- | --- | --- | --- | --- |
| rs4149015 | SLCO1B1 c.-11187G>A | 12 | 21283322 | 0.0001 |
| rs1060253 | SLC7A5 c.*438C>G | 16 | 87866138 | 0.0007 |
| rs4149117 | SLCO1B3 c.334G>T(A112S) | 12 | 21011480 | 0.0015 |
| rs7311358 | SLCO1B3 c.699A>G(I233M) | 12 | 21015760 | 0.0015 |
| rs2053098 | SLCO1B3 c.1557G>A  (A519A) | 12 | 21036411 | 0.0015 |
| rs4149056 | SLCO1B1 c.521T>C  (V174A) | 12 | 21331549 | 0.0081 |
| rs324420 | FAAH c.385C>A(P129T) | 1 | 46870761 | 0.0199 |
| rs10868153 | SLC28A3 c.-47A>C | 9 | 86983368 | 0.0278 |
| rs1565814 | CHST11 c.*1379A>G | 12 | 105152960 | 0.0326 |
| rs6965343 | POR c.189-8806C>T | 7 | 75592925 | 0.0326 |
| rs6664 | CHST2 c.2082C>T | 3 | 142841740 | 0.0349 |
| rs3856806 | PPARG c.1431C>T(H477H) | 3 | 12475557 | 0.0372 |
| rs13959 | ALDH1A1 c.225C>T(S75S) | 9 | 75545882 | 0.0393 |
| rs4148945 | CHST3 c.*1361C>T | 10 | 73769590 | 0.0397 |
| rs7512729 | CYP4Z1 c.1202-2730A>C | 1 | 47578471 | 0.0420 |
| rs7636910 | ABCC5 c.1146A>G(Q382Q) | 3 | 183699516 | 0.0426 |
