# Supplementary material for: Pharmacogenomic Study Reveals New Variants of Drug Metabolizing Enzyme and Transporter Genes Associated with Steady-State Plasma Concentrations of Risperidone and 9-Hydroxyrisperidone in Thai Autism Spectrum Disorder Patients
Source: Front Pharmacol. 2016 Dec 2;7:475. doi: 10.3389/fphar.2016.00475 (PMC5147413; doi:10.3389/fphar.2016.00475)
Supplement: Supplementary file 5 [file Table_4.DOCX]

**Supplementary Table S4**. Top SNPs associated with risperidone/9-hydroxyrisperidone metabolic ratio (Sample size = 102); *P*<0.05

| SNP rsID | Marker name | Chromosome | Marker position | *P* values |
| --- | --- | --- | --- | --- |
| rs1131878 | UGT2B4 c.*448A>G | 4 | 70345904 | 0.0022 |
| rs1058164 | CYP2D6 c.1661G>C(V136V) | 22 | 42525132 | 0.0030 |
| rs1135840 | CYP2D6 c.4180G>C(S486T) | 22 | 42522613 | 0.0046 |
| rs28360521 | CYP2D6 c.-2178G>A | 22 | 42528976 | 0.0050 |
| rs11249454 | UGT2A1 c.715+13414A>G | 4 | 70499234 | 0.0166 |
| rs1881668 | SULT1E1 c.-10+311G>C | 4 | 70725456 | 0.0170 |
| rs1801243 | ATP7B c.1216T>G(S406A) | 13 | 52548140 | 0.0180 |
| rs1045642 | ABCB1 c.3435C>T(I1145I) | 7 | 87138645 | 0.0196 |
| rs4646227 | SLC15A1 c.1256G>C(G419A) | 13 | 99358401 | 0.0228 |
| rs11568482 | SLC22A8 c.913A>T(I305F) | 11 | 62763264 | 0.0335 |
| rs7987433 | SLC10A2 c.-457A>G | 13 | 103719056 | 0.0354 |
| rs971074 | ADH7 c.690G>A(R230R) | 4 | 100341861 | 0.0376 |
| rs1442477 | ADH7 c.-5360G>A | 4 | 100361787 | 0.0376 |
| rs3822172 | SULT1E1 c.-9-469A>G | 4 | 70723840 | 0.0411 |
| rs1126692 | FMO1 c.1188A>G(V396V) | 1 | 171252287 | 0.0495 |
